# Comparative Study of Plasticized Polyvinyl Alcohol and Hydroxypropyl Methylcellulose Films for Pharmaceutical Applications: Mechanical, Thermal, Structural and Disintegration Properties

**DOI:** 10.3390/polym18101211

**Published:** 2026-05-16

**Authors:** Rittin Abraham Kurien, Gokul Kannan, Wantanwa Krongrawa, Supakij Suttiruengwong, Pornsak Sriamornsak

**Affiliations:** 1 Faculty of Pharmacy, Silpakorn University, Nakhon Pathom 73000, Thailand; rittin.ak@saintgits.org (R.A.K.); krongrawa_w@su.ac.th (W.K.); 2 Sustainable Materials Laboratory, Department of Materials Science and Engineering, Faculty of Engineering and Industrial Technology, Silpakorn University, Nakhon Pathom 73000, Thailand; gokul.k@eec.srmrmp.edu.in (G.K.); suttiruengwong_s@su.ac.th (S.S.); 3 Department of Mechanical Engineering, Saintgits College of Engineering (Autonomous), Kottayam 686532, Kerala, India; 4 Center for Material Science, Easwari Engineering College, Chennai 600089, Tamil Nadu, India; 5 Center for Research, SRM TRP Engineering College, Tiruchirappalli 621105, Tamil Nadu, India; 6 Academy of Science, The Royal Society of Thailand, Bangkok 10300, Thailand

**Keywords:** hydroxypropyl methylcellulose, polyvinyl alcohol, plasticizer, polymeric film, crystallography

## Abstract

Polyvinyl alcohol (PVA) and hydroxypropyl methylcellulose (HPMC) films plasticized with glycerin or polyethylene glycol (PEG) were investigated to elucidate structure–property relationships in hydrophilic polymeric film systems. Films were prepared by solution casting at a fixed polymer concentration of 2.7% *w*/*w* with plasticizer contents ranging from 0.49 to 1.33% *w*/*w*, yielding continuous, free-standing films with good surface integrity. Polymer type and plasticizer dosage strongly affected film breakdown behavior. HPMC films with high plasticization swelled and disintegrated. Effective plasticization was shown by a steady drop in tensile strength and elastic modulus and a significant rise in elongation at break. PVA films plasticized better than HPMC films in PEG-containing solutions. Fourier transform infrared spectroscopy verified hydrogen bonding-driven polymer–plasticizer interactions, with glycerin outperforming PEG. Increasing plasticizer percentage reduced crystallographic order and thermal transition temperature in X-ray diffraction and differential scanning calorimetry. Scanning electron microscopy indicated smooth and uniform surfaces at intermediate plasticizer levels, but variability at higher loadings. Among the studied formulations, PVA films containing 1.33% *w*/*w* plasticizer and HPMC films containing 1.05% *w*/*w* plasticizer provided the most balanced combination. These findings support physiochemically rational PVA and HPMC film design for pharmaceutical applications.

## 1. Introduction

Polyvinyl alcohol (PVA) and hydroxypropyl methylcellulose (HPMC) are among the most widely used synthetic and semi-synthetic polymers for the fabrication of thin, flexible films in a broad range of industrial applications, including food packaging, coatings, pharmaceutical products, and functional polymer systems [1]. Their popularity arises from a combination of desirable attributes, such as excellent film-forming ability, hydrophilicity, mechanical tunability, and compatibility with aqueous processing routes. These characteristics make PVA and HPMC particularly attractive for applications where uniformity, flexibility, and processability are essential. In the pharmaceutical field, these polymers are also widely explored as film-forming matrices for oral and transdermal drug delivery systems, particularly fast-dissolving films designed to improve patient compliance and dosing convenience. Despite their widespread use, differences in molecular structure and physical behavior between these polymers continue to motivate fundamental studies aimed at understanding how formulation variables govern film performance.

In particular, PVA is recognized for its semicrystalline structure, relatively high tensile strength, and strong intermolecular hydrogen-bonding capability [2]. These features contribute to the formation of films with good mechanical integrity and cohesive strength. Compared to PVA, HPMC has a non-crystalline structure, strong thermal stability, and good rheological behavior, resulting in films with lower tensile strength but higher flexibility and hydration and swelling. Such intrinsic differences make PVA and HPMC attractive model polymers for investigating structure–property relationships in plasticized polymeric films, especially when examined under comparable processing conditions. These differences are particularly relevant in potential drug delivery applications, where both mechanical integrity during handling and rapid hydration or disintegration are critical performance requirements.

The molecular structure of PVA contains abundant hydroxyl groups along the polymer backbone, which promote extensive intermolecular hydrogen bonding and facilitate interactions with both inorganic and organic nanomaterials. This capability has enabled the development of nanocomposites and multifunctional materials with enhanced mechanical, barrier, or surface properties [3]. Consequently, numerous studies have focused on tailoring the physicochemical and structural properties of PVA films, with particular attention to crystallization behavior, molecular organization, and phase transitions [4,5]. Beyond crystallinity control, a variety of material engineering strategies—including surface chemical treatments, polymer blending, and incorporation of nanocomposites—have been explored to develop advanced barrier materials and to improve the mechanical and transport properties of PVA-based films [6,7,8]. As a result, PVA readily forms strong, flexible, and transparent films with excellent tensile strength and barrier performance, making it suitable for packaging and coating applications. Its high water-solubility further facilitates processing and formulation across multiple industries [9], while also providing strong bonding strength to substrates such as paper and wood.

HPMC is similarly valued for its good film-forming capability and broad acceptability across various applications [10]. It functions as an effective thickener and stabilizer and is widely employed in products ranging from pharmaceuticals to construction materials due to its favorable rheological properties. From a structural perspective, the substituted cellulose backbone of HPMC leads to lower crystallinity and distinct hydration behavior compared with PVA. These differences influence film flexibility, moisture sensitivity, and mechanical response, highlighting the importance of polymer chemistry in governing macroscopic film properties. Together, PVA and HPMC offer complementary characteristics that are well suited for comparative investigations of polymeric film systems.

When formulating PVA or HPMC films, several key factors must be considered, among which the incorporation of plasticizers plays a central role. Plasticizers such as glycerin are commonly used to enhance flexibility and reduce brittleness by increasing polymer chain mobility and weakening intermolecular interactions [11]. The type and concentration of plasticizer significantly affect not only mechanical properties, but also thermal behavior and hydration-related responses of the resulting films. In addition to plasticizers, various additives may be incorporated to impart specific functionalities, including antimicrobial activity or colorimetric responses for smart packaging applications [12]. Despite extensive use of plasticized PVA and HPMC films, the combined effects of polymer composition and plasticizer chemistry on overall film performance remain insufficiently understood. In the context of potential drug delivery applications, controlling these parameters is essential for achieving a balance between mechanical robustness and rapid disintegration or disintegration behavior.

Therefore, in this study, the influence of plasticizer content on the mechanical performance, structural organization, thermal behavior, and aqueous disintegration characteristics of PVA and HPMC films was systematically investigated. Through this approach, clearer insight into structure–property relationships in plasticized polymeric films could be obtained, providing a scientific basis for the rational design and optimization of flexible and functional polymeric materials for diverse engineering applications. The findings are intended to support the early-stage development of pharmaceutical applications.

## 2. Materials and Methods

### 2.1. Materials

PVA (weight-average molecular weight (Mw) 124,000–186,000 Da, ≥99% hydrolyzed) was purchased from Sigma-Aldrich (St. Louis, MO, USA). HPMC (HPMC E15; pharmaceutical grade, low-viscosity) was obtained from Meska JoinWay Pharmaceutical Co., Ltd. (Huzhou, China). The material corresponds to a nominal viscosity grade of 15 cP (2% aqueous solution at 20 °C) and typically possesses a Mw in the range of approximately 20,000–40,000 Da. Glycerin was supplied by Ajax Finechem Pvt. Ltd. (Auckland, New Zealand), and PEG was purchased from Scharlau Chemie S.A. (Barcelona, Spain). Distilled water was used as the solvent for all film preparations.

### 2.2. Film Preparation

Polymeric films were prepared using a solution-casting technique. Four film systems were fabricated: PVA–glycerin, PVA–polyethylene glycol, HPMC–glycerin, and HPMC–polyethylene glycol. For PVA–glycerin films, PVA was initially dispersed in distilled water at a concentration of 3.0% *w*/*w* in a cylindrical flask. The dispersion was heated and stirred on a magnetic hotplate at 80 °C until complete dispersion was achieved and a clear, homogeneous solution was obtained. Glycerin was then added to the polymer solution at final concentrations of 0.49, 0.78, 1.05, and 1.33% *w*/*w*, after which the effective polymer concentration in the mixture became 2.7% *w*/*w*. The resulting solution was continuously stirred at 80 °C for 1 h to ensure uniform distribution of the plasticizer. The resulting homogeneous solution was cast onto an acrylic rectangular mold (270 mm × 130 mm) to form thin films. Solvent evaporation was carried out in a hot-air oven at 45 °C for 18 h prior to characterization. Four sets of films were prepared for each polymer–plasticizer combination corresponding to the different plasticizer concentrations. The thickness of the film was ≈0.09 mm.

**Figure 1:**
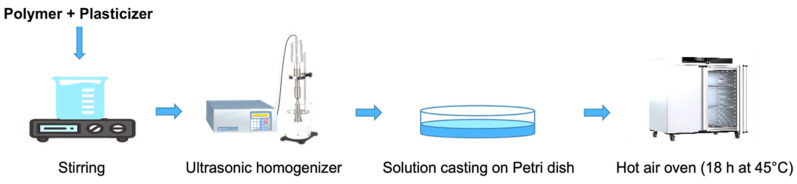
Schematic representation of film preparation.

The same preparation procedure was applied for PVA–PEG, HPMC–glycerin, and HPMC–PEG films. In all formulations, the final polymer concentration (before drying) was fixed at 2.7% *w*/*w*, while the plasticizer content varied from 0.49 to 1.33% *w*/*w*. The compositions of the prepared films are summarized in Table 1. A schematic illustration of the film preparation process is presented in Figure 1.

### 2.3. Film Disintegration Test

**Figure 2:**
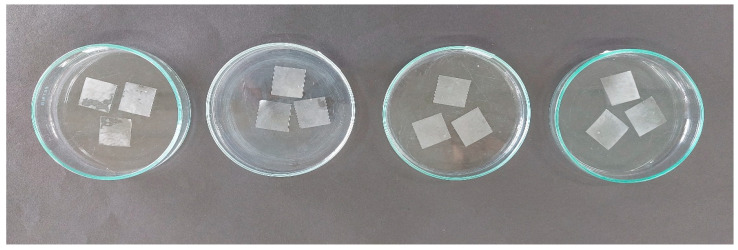
Samples prepared for film disintegration test.

Film disintegration behavior was evaluated to assess the hydration and disintegration characteristics of the prepared polymeric films. Square film specimens (2 cm × 2 cm) were immersed in 150 mL of distilled water maintained at 37 ± 0.5 °C, under static conditions to simulate the conditions in the oral cavity during the disintegration of oral films. The time required for complete film disintegration was recorded using a stopwatch. All measurements were conducted in triplicate. Representative samples prepared for the disintegration test are shown in Figure 2.

### 2.4. Film Characterization

Due to biodegradability, flexibility and compatibility with active compounds, plasticized PVA and HPMC films are popular in sustainable packaging and biomedical applications. Characterizing these films’ structure-property interactions is necessary to optimize their performance. This investigates the films’ mechanical, chemical, and morphological properties to understand how plasticizers affect tensile strength, chemical stability and microstructure.

#### 2.4.1. Mechanical Properties

Tensile strength, tensile modulus, and elongation at break were measured using a TA.XT Plus texture analyzer (Stable Micro Systems, Godalming, UK) at ambient temperature (25 °C). Prior to testing, the films were conditioned in a desiccator for at least 48 h. Rectangular specimens with dimensions of 100 mm × 25 mm were cut from each film. At least five replicate measurements were performed for each formulation, and the results were reported as mean values with corresponding standard deviations.

#### 2.4.2. Fourier Transform Infrared Spectroscopy (FTIR)

FTIR spectra were collected using an Alpha II FTIR spectrometer (Bruker Optics GmbH, Ettlingen, Germany). Spectra were recorded in the range of 4000–500 cm^−1^ at a resolution of 4 cm^−1^, with 32 scans accumulated for each film sample.

#### 2.4.3. X-Ray Diffraction (XRD)

X-ray diffraction patterns were obtained using a D/max-rB X-ray diffractometer equipped with Cu Kα radiation. Samples were scanned over a 2θ range of 10–80° at a scanning rate of 4°/min to analyze crystalline structure and phase behavior.

#### 2.4.4. Differential Scanning Calorimetry (DSC)

Thermal properties of the films were analyzed using a DSC Q100 (TA Instruments, New Castle, DE, USA; Version 9.9 Build 303). Approximately 2–3 mg of each film sample was sealed in an aluminum pan and heated from −90 to 400 °C at a heating rate of 20 °C/min under a nitrogen atmosphere with a flow rate of 50 mL/min. Glass transition temperature, melting temperature, and thermal degradation behavior were determined from the DSC thermograms.

#### 2.4.5. Morphological Characterization

Surface morphology of the films was examined using field-emission scanning electron microscopy (FESEM; Tescan Mira 3, Brno, Czech Republic). Film samples were air-dried, fractured, and sputter-coated with a thin layer of gold prior to observation. FESEM micrographs were used to assess surface uniformity, homogeneity, and morphological changes induced by plasticizer incorporation.

### 2.5. Statistical Analysis

All experiments were performed in triplicate or as otherwise specified, and the results are expressed as mean ± standard deviation (SD). The mean values and standard deviations were calculated using Microsoft Excel (Microsoft Corporation, Redmond, WA, USA).

## 3. Results and Discussion

### 3.1. Film Preparation and Visual Appearance

**Figure 3:**
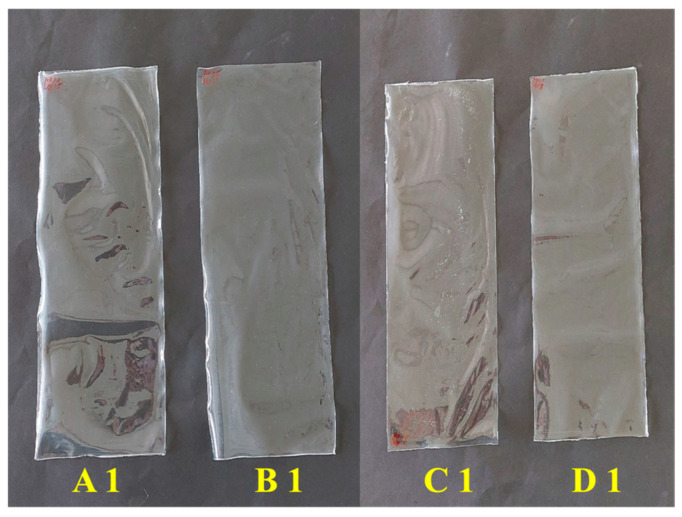
Prepared film samples: (**A1**) PVA–glycerin, (**B1**) PVA–polyethylene glycol, (**C1**) HPMC–glycerin, and (**D1**) HPMC–polyethylene glycol.

All polymeric films were successfully prepared using the solution-casting method, producing continuous, free-standing films without visible cracks or obvious defects. In all formulations, the final polymer concentration was fixed at 2.7% *w*/*w*, while the final plasticizer content ranged from 0.49 to 1.33% *w*/*w*. Representative images of the fabricated films are presented in Figure 3. The films could be readily removed from the casting substrate without tearing, indicating sufficient film integrity and cohesive strength across all compositions.

From visual inspection, the films were generally smooth and flexible, with their appearance ranging from transparent to slightly translucent depending on the polymer type and plasticizer composition. PVA-based films were typically more transparent and exhibited more uniform surfaces, which can be attributed to the semicrystalline nature of PVA and its tendency to form a compact hydrogen-bonded network during film formation. In contrast, films prepared from HPMC appeared somewhat less transparent and displayed a softer surface texture, consistent with the more amorphous structure of HPMC and its greater affinity for water.

Plasticizer content also had a noticeable effect on film appearance and handling characteristics. Films containing lower plasticizer levels were relatively stiff, whereas those prepared with higher amounts of glycerin or polyethylene glycol were more flexible and easier to handle. The surfaces of these films appeared smoother, suggesting enhanced polymer chain mobility resulting from effective plasticization. No visible phase separation, plasticizer migration, or surface crystallization was detected across the investigated composition range, indicating good compatibility between the polymers and plasticizers. Minor surface irregularities were occasionally observed, likely arising from solvent evaporation during the casting process, but these did not adversely affect film integrity. Taken together, these visual observations support the suitability of the selected preparation conditions for producing uniform polymeric films and provide a useful qualitative foundation for the subsequent physicochemical characterization.

### 3.2. Film Disintegration Test

The disintegration behavior of the polymeric films is summarized in Table 2, where clear differences can be observed as a function of polymer type, plasticizer type, and plasticizer concentration. PVA-based films exhibited significantly shorter disintegration times than HPMC-based films, reflecting inherent differences in polymer structure, crystallinity, and hydration behavior.

Among all tested samples, sample B4 (PVA plasticized with 1.33% *w*/*w* PEG) showed the shortest disintegration time of 4.7 s. For PVA–PEG films, a consistent decrease in disintegration time was observed with increasing PEG content. This trend indicates that higher plasticizer concentrations promote faster hydration and disintegration of the film matrix. PEG, being a low-molecular-weight, highly hydrophilic plasticizer, increases the free volume within the polymer network and weakens intermolecular hydrogen bonding between PVA chains [13]. Consequently, polymer chain mobility is enhanced, leading to a less compact structure that allows rapid penetration of water into the film and accelerates disintegration.

A similar, though less pronounced, trend was observed for PVA films plasticized with glycerin. Increasing glycerin concentration reduced disintegration time from 24 s at 0.49% *w*/*w* to 12.67 s at 1.33% *w*/*w*. The slower disintegration of glycerin-plasticized films compared with PEG-plasticized films may be attributed to differences in plasticizer molecular size and interaction strength with PVA. Glycerin can form strong hydrogen bonds with hydroxyl groups along the PVA backbone, which may partially limit water diffusion despite increased chain flexibility.

In contrast to PVA systems, HPMC-based films exhibited substantially longer disintegration times, ranging from approximately 62 to 127 s depending on formulation. This behavior can be linked to the predominantly amorphous nature of HPMC and its tendency to swell and form a hydrated gel layer upon contact with water. Such swelling can initially retard complete film disintegration by creating a viscous diffusion barrier, particularly at lower plasticizer concentrations [14].

For HPMC films plasticized with glycerin, the disintegration time decreased as the plasticizer content increased up to 1.05% *w*/*w*, but a slight increase was observed at 1.33% *w*/*w*. A comparable non-monotonic trend was noted for HPMC–polyethylene glycol films, where the fastest disintegration occurred at 1.05% *w*/*w* PEG, followed by a modest increase at higher plasticizer loading. This behavior suggests that excessive plasticizer addition may increase polymer chain mobility to a point where film swelling and structural relaxation become dominant, rather than promoting rapid film breakup. As a result, the overall disintegration process may be prolonged. Similar effects have been reported for hydrophilic polymer systems in which competing interactions between polymer chains, plasticizer molecules, and water govern hydration behavior. FTIR research demonstrates that elevating plasticizer content reduces hydrogen bonding intensity (e.g., broadening and shifting of O–H stretching bands), disrupting intermolecular connections. DSC studies show that plasticization increases polymer chain mobility by lowering glass transition temperature (T_g_). XRD patterns show reduced crystallinity (increased peak broadening/lower intensity), indicating an improved amorphous polymer network.

These observations indicate that film disintegration is controlled by a balance between polymer chemistry, plasticizer type, and plasticizer concentration. Although increasing plasticizer content generally enhances chain mobility and water uptake, leading to faster disintegration, excessive plasticization, particularly in highly hydrophilic polymers such as HPMC can shift the mechanism toward swelling-controlled behavior that delays complete disintegration [15]. This highlights the need to carefully optimize plasticizer content to achieve the desired aqueous response in polymeric film systems.

### 3.3. Mechanical Properties

**Figure 4:**
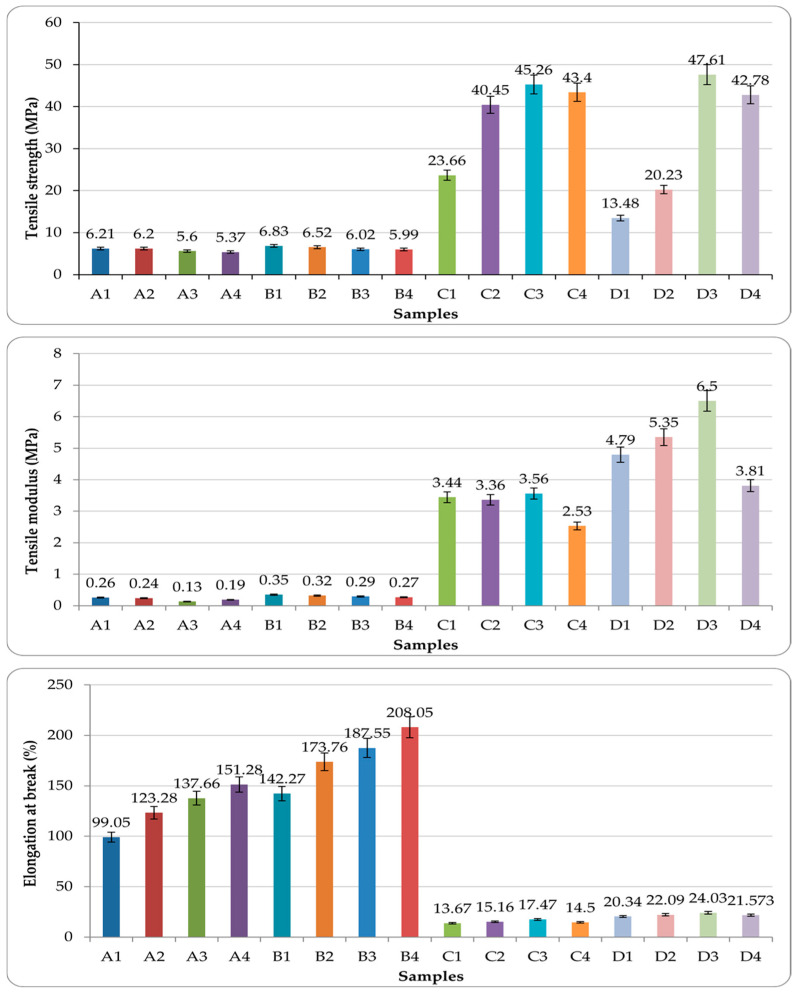
Mechanical properties of fabricated films (mean ± standard deviation, n = 5).

The mechanical properties of the fabricated films, including tensile strength, tensile modulus, and elongation at break, are summarized in Figure 4. Clear differences were observed depending on polymer type, plasticizer type, and plasticizer concentration, highlighting the strong influence of plasticization on film mechanical behavior.

For PVA-based films plasticized with either glycerin or PEG, a clear trend was observed in which increasing plasticizer content led to a gradual reduction in tensile strength and tensile modulus, together with a marked increase in elongation at break. This response is typical of effective plasticization, where low-molecular-weight plasticizers weaken intermolecular interactions between polymer chains and enhance chain mobility. Among the PVA formulations, sample B4 (PVA containing 1.33% *w*/*w* PEG) exhibited the highest elongation at break (approximately 208%), indicating the formation of a highly flexible film. At comparable concentrations, PEG-plasticized PVA films consistently showed greater extensibility than those plasticized with glycerin, suggesting that PEG provides a more efficient plasticizing effect within the PVA matrix.

The observed decrease in stiffness and strength with increasing plasticizer content can be attributed to disruption of polymer–polymer hydrogen bonding and an increase in free volume within the polymer network. Glycerin and PEG molecules, owing to their relatively small molecular size and the presence of hydroxyl functional groups, can penetrate between adjacent polymer chains and form polymer–plasticizer interactions that reduce cohesive forces between macromolecules [16,17,18]. This molecular rearrangement facilitates chain slippage under applied stress, leading to enhanced ductility at the expense of resistance to deformation.

In contrast to PVA systems, HPMC-based films exhibited substantially higher tensile strength and tensile modulus but very limited elongation at break, reflecting their inherently stiffer and more brittle character [19]. At lower plasticizer concentrations, HPMC films showed minimal extensibility, with elongation at break values below 5%, which is consistent with the rigid cellulose-derived backbone of HPMC and the strong intermolecular interactions within its polymer network. The incorporation of glycerin or PEG influenced the mechanical behavior of HPMC films in a more complex and non-linear manner. Tensile strength and modulus increased with plasticizer content up to an intermediate concentration of 1.05% *w*/*w*, after which both parameters declined at higher plasticizer levels. This trend suggests that moderate plasticization may enhance stress distribution and packing efficiency within the HPMC matrix, whereas excessive plasticizer addition leads to over-plasticization and a partial loss of structural cohesion.

HPMC films plasticized with PEG generally exhibited higher elongation at break than those containing glycerin, particularly at intermediate plasticizer concentrations. This difference may be associated with variations in molecular size and flexibility between polyethylene glycol and glycerin, which influence chain mobility and the balance between plasticization and network integrity [20]. At higher plasticizer contents, however, elongation values decreased, indicating that excessive plasticizer incorporation may promote the formation of localized soft domains that act as stress concentrators during tensile deformation.

The mechanical results indicate that plasticizer type and concentration strongly influence the balance between strength and flexibility in both PVA and HPMC film systems. PVA films readily develop high ductility as plasticizer content increases, whereas HPMC films exhibit a narrower compositional range in which mechanical performance can be effectively enhanced. This difference reflects the inherent structural rigidity of the HPMC backbone and its limited tolerance to plasticization. These findings emphasize the need for careful optimization of plasticizer loading to achieve targeted mechanical properties in polymeric films designed for specific performance requirements.

### 3.4. FTIR Analysis

**Figure 5:**
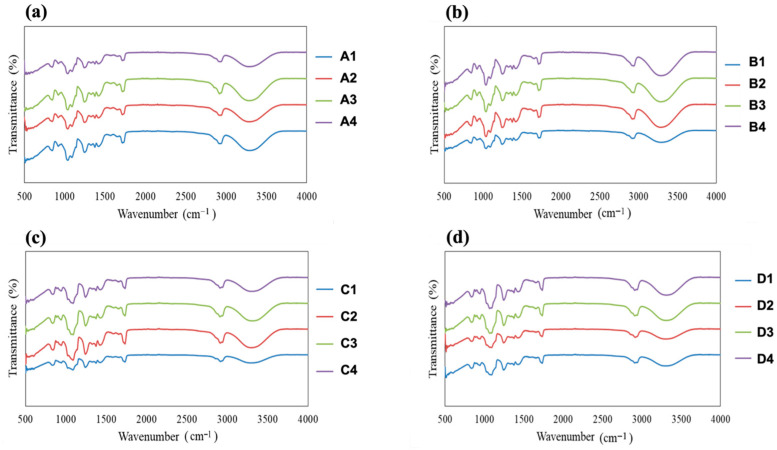
FTIR spectra of fabricated film samples: (**a**) PVA–glycerin; (**b**) PVA–PEG; (**c**) HPMC–glycerin; and (**d**) HPMC–PEG.

The FTIR spectra of all fabricated films are presented in Figure 5. Comparative analysis of the spectra provides insight into the molecular interactions between the polymer matrices (PVA or HPMC) and the plasticizers (glycerin or PEG) as a function of plasticizer concentration. All spectra exhibit a broad absorption band in the range of 3200–3400 cm^−1^, which is attributed to O–H stretching vibrations arising from hydroxyl groups present in PVA, HPMC, and the polyol plasticizers. The breadth and intensity of this band increased with increasing plasticizer concentration, indicating enhanced hydrogen-bonding interactions between the polymer chains and plasticizer molecules. Bands observed in the 2800–3000 cm^−1^ region correspond to C–H stretching vibrations from aliphatic groups in both the polymers and plasticizers. The fingerprint region between 500 and 1500 cm^−1^ contains overlapping contributions from C–O stretching, C–C stretching, and C–H bending vibrations. Subtle changes in peak shape, intensity, and broadening within this region were observed as a function of plasticizer type and concentration, reflecting modifications in local chain environment and molecular packing induced by plasticization.

Comparing the two polymer systems, PVA-based films generally exhibited sharper O–H and C–H stretching bands, suggesting a more organized hydrogen-bonding network within the semicrystalline PVA matrix. In contrast, HPMC-based films showed broader and less well-defined absorption bands, consistent with the predominantly amorphous structure of HPMC and a less rigid hydrogen-bonding framework. This difference indicates that plasticizers may be more uniformly incorporated into the HPMC matrix, leading to greater spectral broadening. Differences were also evident between glycerin- and PEG-plasticized films. Glycerin-containing films displayed a more pronounced increase in O–H band intensity with rising plasticizer concentration, reflecting the presence of three hydroxyl groups per glycerin molecule and its strong ability to form hydrogen bonds with the polymer chains. In contrast, PEG-plasticized films showed comparatively lower O–H band intensity, suggesting weaker or more distributed hydrogen-bonding interactions between PEG and the polymer matrices.

With increasing plasticizer content from 0.49 to 1.33% *w*/*w*, all film systems exhibited progressive band broadening and increased intensity in both the O–H and C–O stretching regions. These spectral changes indicate increased polymer–plasticizer interactions, enhanced chain mobility, and a higher degree of structural disorder within the films. Minor peak shifts and broadening observed in the fingerprint region further support a reduction in intermolecular order and possible decreases in crystallinity at higher plasticizer levels. The FTIR results clearly demonstrate that both plasticizer type and concentration strongly influence molecular interactions within PVA and HPMC films. Glycerin produces a more pronounced hydrogen-bonding effect than PEG, while HPMC matrices exhibit broader spectral features than PVA, indicating differences in plasticizer incorporation and polymer–plasticizer compatibility. These molecular-level insights are consistent with the observed changes in mechanical behavior and disintegration characteristics discussed in previous sections.

### 3.5. XRD Analysis

**Figure 6:**
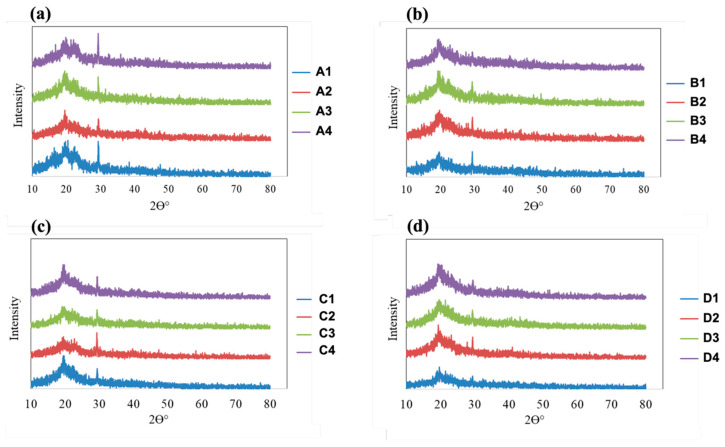
XRD patterns of fabricated film samples: (**a**) PVA–glycerin; (**b**) PVA–PEG; (**c**) HPMC–glycerin; and (**d**) HPMC–PEG.

The XRD patterns of all fabricated films are presented in Figure 6. The diffraction profiles provide insight into changes in the crystalline structure of PVA- and HPMC-based films as a function of plasticizer type and concentration. All samples exhibited a broad diffraction peak in the range of 2θ ≈ 19–23°, which is characteristic of semicrystalline polymer systems such as PVA and cellulose derivatives. This broad reflection arises from short-range ordered regions embedded within an amorphous matrix. As the concentration of either PEG or glycerin increased, a gradual reduction in peak intensity and sharpness was observed, accompanied by peak broadening and an elevated baseline. These changes indicate a progressive decrease in crystalline order and an increase in amorphous content within the films.

For PVA-based films (samples A1–A4 and B1–B4), increasing plasticizer concentration led to a noticeable weakening and broadening of the main diffraction peak. This behavior suggests that both PEG and glycerin disrupt the regular packing of PVA chains by inserting between polymer segments and interfering with intermolecular hydrogen bonding. As a result, the formation of ordered crystalline domains is suppressed, leading to a more amorphous and flexible polymer network. A similar trend was observed for HPMC-based films (samples C1–C4 and D1–D4), although the diffraction peaks were broader and less intense even at the lowest plasticizer concentration. This observation is consistent with the inherently amorphous nature of HPMC. With increasing plasticizer content, further broadening of the diffraction profiles occurred, indicating an enhanced disruption of residual ordered regions and a transition toward a predominantly amorphous structure.

When comparing plasticizer types, glycerin generally produced a more pronounced reduction in peak intensity and greater peak broadening than PEG at comparable concentrations. This effect can be attributed to the smaller molecular size of glycerin and the presence of multiple hydroxyl groups, which promote strong hydrogen-bonding interactions with the polymer chains and more effectively disrupt chain packing. The effect of glycerin was particularly evident in HPMC-based films, where crystallinity was reduced to a greater extent than in corresponding PEG-plasticized samples. Increasing the plasticizer concentration from 0.49 to 1.33% *w*/*w* resulted in a systematic decrease in crystalline order for both polymer systems. Films containing the highest plasticizer levels exhibited the broadest and least intense diffraction peaks, confirming a substantial loss of crystalline domains. These structural changes observed by XRD are consistent with the enhanced flexibility, stretchability, and optical clarity typically associated with highly plasticized, amorphous polymeric films. The XRD results therefore confirm that both PEG and glycerin act as effective plasticizers in PVA and HPMC matrices, with glycerin showing a stronger influence on reducing crystallinity.

### 3.6. DSC Analysis

**Figure 7:**
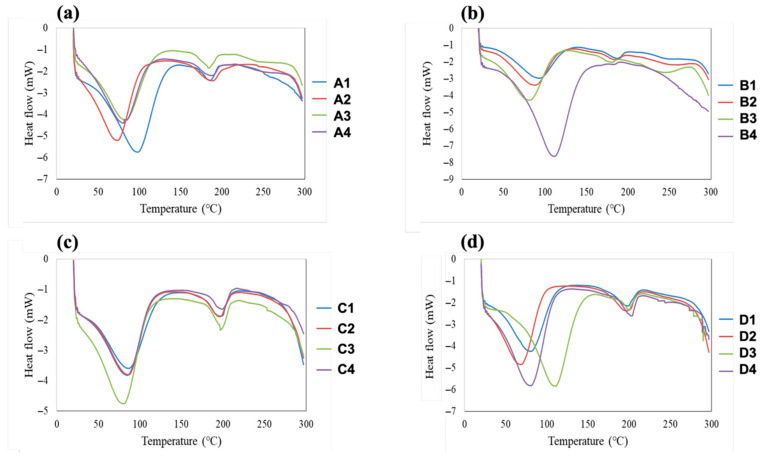
DSC thermograms of fabricated film samples: (**a**) PVA–glycerin; (**b**) PVA–PEG; (**c**) HPMC–glycerin; and (**d**) HPMC–PEG.

The DSC thermograms of all fabricated films are shown in Figure 7. These thermograms provide insight into the influence of plasticizer type and concentration on the thermal behavior of PVA and HPMC films. Across all samples, thermal transitions were observed within the temperature range of approximately 40–300 °C, indicating plasticizer-induced modifications in polymer chain mobility and structural organization. With increasing plasticizer concentration, the major transition peaks generally shifted toward lower temperatures and became broader and less intense. Such behavior is characteristic of effective plasticization and reflects reduced crystallinity and enhanced molecular mobility within the polymer matrix.

For PVA-based films (samples A1–A4 and B1–B4), the addition of glycerin or PEG resulted in a progressive decrease in the temperatures of the main thermal transitions. The transition observed around ~100 °C, often associated with the glass transition or relaxation of amorphous regions in PVA, shifted to lower temperatures and became less distinct as plasticizer content increased. At higher plasticizer levels, particularly at 1.33% *w*/*w*, this transition was significantly weakened or nearly disappeared, indicating extensive disruption of ordered domains and substantial plasticization. Glycerin produced more pronounced peak broadening and downward shifts than PEG, suggesting a stronger interaction with PVA chains. In HPMC-based films (samples C1–C4 and D1–D4), similar trends were observed, although the thermal transitions were generally broader and occurred at slightly lower temperatures compared with PVA films. This behavior is consistent with the more amorphous nature of HPMC. Increasing plasticizer content led to further broadening and attenuation of thermal transitions, particularly in the 50–120 °C region, confirming an increase in amorphous character and reduced intermolecular order. As observed for PVA systems, glycerin exerted a stronger plasticizing effect than PEG, producing greater reductions in Tg and more diffuse thermal events, especially at higher plasticizer concentrations (1.05 and 1.33% *w*/*w*).

Comparatively, PEG also lowered Tg and Tm values but to a lesser extent than glycerin. The transitions in PEG-plasticized films remained relatively sharper, suggesting partial retention of ordered or semicrystalline regions within the polymer matrix. This difference can be attributed to the molecular size and flexibility of PEG, which may lead to more distributed polymer–plasticizer interactions without fully disrupting crystalline packing. In short, increasing plasticizer content from 0.49 to 1.33% *w*/*w* resulted in systematic lowering of thermal transition temperatures, broadening of DSC peaks, and reduced transition enthalpy for both polymer systems. These effects were more pronounced for glycerin than PEG and more evident in HPMC than in PVA films. The DSC results clearly confirm the plasticizing role of both glycerin and PEG, with glycerin acting as a more efficient plasticizer due to its stronger disruption of intermolecular forces and crystalline domains. The observed thermal behavior is consistent with the structural changes identified by XRD and the molecular interactions revealed by FTIR analysis, collectively indicating that increased plasticizer content—particularly glycerin—leads to enhanced chain mobility, reduced crystallinity, and a more amorphous polymer network.

### 3.7. SEM Analysis

**Figure 8:**
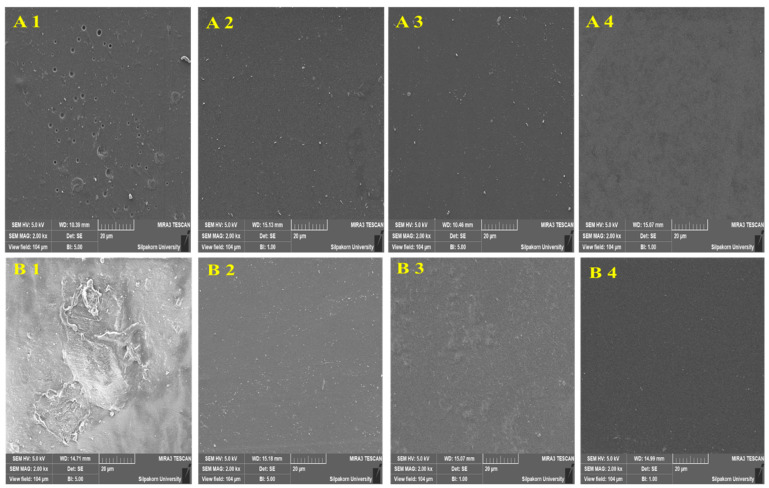

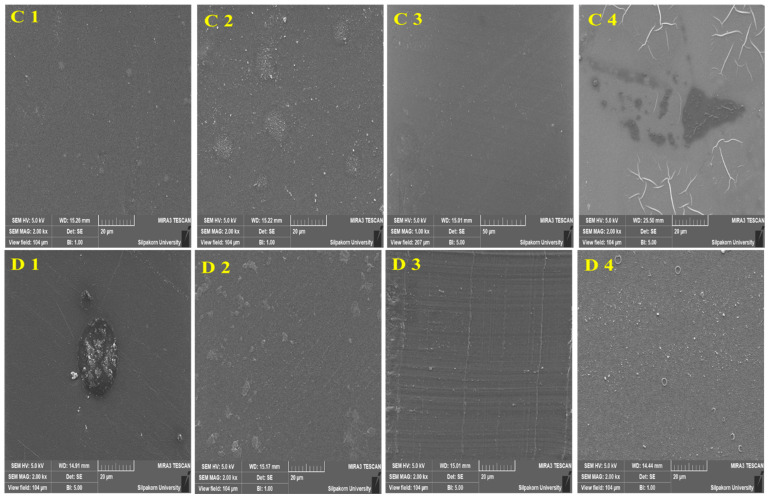
Scanning electron microscopy (SEM) images of fabricated film surfaces. The films were prepared using the following formulations: (**A1**–**A4**) 2.7% *w*/*w* PVA with 0.49%, 0.78%, 1.05%, and 1.33% *w*/*w* glycerin, respectively; (**B1**–**B4**) 2.7% *w*/*w* PVA with 0.49%, 0.78%, 1.05%, and 1.33% *w*/*w* PEG, respectively; (**C1**–**C4**) 2.7% *w*/*w* HPMC with 0.49%, 0.78%, 1.05%, and 1.33% *w*/*w* glycerin, respectively; and (**D1**–**D4**) 2.7% *w*/*w* HPMC with 0.49%, 0.78%, 1.05%, and 1.33% *w*/*w* PEG, respectively.

SEM was employed to examine the surface morphology of the fabricated polymeric films, and representative micrographs are shown in Figure 8. The images reveal the influence of polymer composition and plasticizer type and concentration on the surface uniformity and microstructural features of the films. For PVA–glycerin films, sample A4 (PVA with 1.33% *w*/*w* glycerin) exhibited a smooth and continuous surface without visible cracks or phase-separated domains. A similarly uniform surface morphology was observed for B4 (PVA with 1.33% *w*/*w* PEG), indicating effective incorporation of PEG within the PVA matrix. These observations suggest good compatibility between PVA and both plasticizers at higher plasticizer concentrations. In the case of HPMC-based films, smooth and homogeneous surfaces were observed for C3 (HPMC with 1.05% *w*/*w* glycerin) and D3 (HPMC with 1.05% *w*/*w* PEG). The absence of visible pores, cracks, or segregated phases in these samples indicates uniform dispersion of the plasticizers within the HPMC matrix. Overall, these formulations displayed well-developed film surfaces with minimal surface irregularities.

Closer examination of the SEM images revealed that, for samples A4, B4, C3, and D3, the plasticizers were well distributed throughout the polymer matrices, resulting in smooth and homogeneous surfaces. No evidence of plasticizer segregation or crystalline aggregation was observed in these films, suggesting favorable polymer–plasticizer compatibility. The uniform morphology can be attributed to similarities in polarity and hydrogen-bonding capability between the polymers and plasticizers, which facilitate effective intermolecular interactions and promote homogeneous blending [21,22,23,24,25]. The incorporation of glycerin or PEG likely increased the free volume within the polymer networks, contributing to improved flexibility and surface smoothness.

In contrast, other formulations exhibited noticeable surface imperfections, including roughness, shallow pits, protrusions, and localized discontinuities. In some samples, particularly those containing higher PEG concentrations in HPMC films (e.g., C4), the surfaces appeared more irregular and showed signs of microcracking and localized plasticizer-rich regions, which may be described as a slight oily appearance. These features became more pronounced with increasing PEG content, suggesting that excessive plasticizer loading can lead to over-plasticization and reduced surface stability.

Taken together, the SEM observations suggest that both glycerin and PEG are compatible plasticizers for PVA and HPMC films when used within an appropriate concentration range. At suitable plasticizer levels, the films exhibit smooth and uniform surfaces, whereas excessive plasticizer content tends to introduce surface irregularities and compromises structural stability. Notably, formulations A4, B4, C3, and D3, which showed the most homogeneous surface morphologies, also performed well in disintegration testing and exhibited high elongation at break. This consistency across different measurements points to a close connection between surface quality, mechanical flexibility, and hydration behavior. These findings emphasize that careful adjustment of glycerin or PEG content is important for controlling film microstructure and achieving a balanced combination of structural integrity and functional performance in PVA and HPMC film systems.

## 4. Limitations

PVA films exhibit good flexibility; however, they may soften and lose structural integrity under humid or moist conditions, which can affect their stability during storage and pharmaceutical use. In contrast, HPMC films generally show good transparency and homogeneity, but insufficient or excessive plasticizer content may lead to brittleness or reduced mechanical stability during handling and application. In some formulations, uneven plasticizer distribution may also contribute to localized weak regions and reduced film uniformity. Excessive plasticizer incorporation can further reduce thermal stability and promote plasticizer migration during storage, potentially altering film performance over time.

In aqueous environments, differences in polymer hydrophilicity and polymer–plasticizer interactions strongly influence film swelling and disintegration behavior. Furthermore, environmental factors such as humidity and mechanical stress may affect the long-term stability and disintegration characteristics of oral thin films. Variability during scale-up from laboratory casting to industrial manufacturing processes may also influence film homogeneity and batch-to-batch reproducibility.

In addition, the present study focused on placebo polymeric films without incorporation of active pharmaceutical ingredients (APIs). The incorporation of APIs may significantly influence film properties, including mechanical strength, thermal behavior, crystallinity, surface morphology, and disintegration performance, depending on the physicochemical characteristics and loading of the drug. Therefore, further studies are necessary to evaluate drug-loaded formulations and optimize polymer–plasticizer–drug interactions for practical pharmaceutical applications.

## 5. Conclusions

This study examined the effects of glycerin and PEG on the disintegration behavior, mechanical properties, thermal characteristics, structure, and surface morphology of PVA and HPMC films prepared by solution casting. Both polymers exhibited good film-forming ability, producing uniform and transparent films within the investigated formulation range. The results demonstrate that increasing plasticizer content enhances polymer chain mobility through hydrogen-bonding interactions, leading to reduced crystallinity, lower thermal transition temperatures, and improved film flexibility. Glycerin showed a stronger plasticizing effect than PEG, particularly in terms of crystallinity reduction and thermal behavior. Mechanical testing revealed a decrease in tensile strength and modulus accompanied by a substantial increase in elongation at break, with PVA films exhibiting greater extensibility and HPMC films showing higher tensile strength.

Structural and morphological analyses confirmed that optimal plasticizer concentrations yield homogeneous film surfaces, whereas excessive plasticizer content leads to surface irregularities and structural instability. Among the studied formulations, PVA films containing 1.33% *w*/*w* plasticizer and HPMC films containing 1.05% *w*/*w* plasticizer provided the most balanced combination of disintegration behavior, mechanical performance, and surface uniformity. These physicochemical characteristics are particularly relevant for potential film-based drug delivery systems, where both mechanical integrity and controlled hydration or disintegration are required.

The present findings provide a useful foundation for the early-stage development of potential fast-dissolving oral film platforms, supporting subsequent incorporation of active pharmaceutical ingredients. In summary, the findings highlight the importance of plasticizer type and concentration in tailoring the properties of PVA and HPMC films and provide practical guidance for the rational design of flexible polymeric films for functional material applications.

## Figures and Tables

**Table 1:** List of polymeric films prepared.

Sample	Material Combination (Before Drying)
A1	2.7% *w*/*w* PVA + 0.49% *w*/*w* glycerin
A2	2.7% *w*/*w* PVA + 0.78% *w*/*w* glycerin
A3	2.7% *w*/*w* PVA + 1.05% *w*/*w* glycerin
A4	2.7% *w*/*w* PVA + 1.33% *w*/*w* glycerin
B1	2.7% *w*/*w* PVA + 0.49% *w*/*w* PEG
B2	2.7% *w*/*w* PVA + 0.78% *w*/*w* PEG
B3	2.7% *w*/*w* PVA + 1.05% *w*/*w* PEG
B4	2.7% *w*/*w* PVA + 1.33% *w*/*w* PEG
C1	2.7% *w*/*w* HPMC + 0.49% *w*/*w* glycerin
C2	2.7% *w*/*w* HPMC + 0.78% *w*/*w* glycerin
C3	2.7% *w*/*w* HPMC + 1.05% *w*/*w* glycerin
C4	2.7% *w*/*w* HPMC + 1.33% *w*/*w* glycerin
D1	2.7% *w*/*w* HPMC + 0.49% *w*/*w* PEG
D2	2.7% *w*/*w* HPMC + 0.78% *w*/*w* PEG
D3	2.7% *w*/*w* HPMC + 1.05% *w*/*w* PEG
D4	2.7% *w*/*w* HPMC + 1.33% *w*/*w* PEG

**Table 2:** Film disintegration of different films containing different types and concentrations of plasticizer (mean ± standard deviation, n = 3).

Sample	Film Dimension (cm^2^)	Film Weight (mg)	Disintegration Time (s)
A1	2 × 2	39.77 ± 0.68	24.00 ± 7.00
A2	2 × 2	40.12 ± 0.21	22.33 ± 4.04
A3	2 × 2	40.78 ± 0.85	20.00 ± 8.72
A4	2 × 2	39.44 ± 0.31	12.67 ± 3.79
B1	2 × 2	40.23 ± 0.36	10.33 ± 0.58
B2	2 × 2	40.89 ± 0.92	7.33 ± 1.15
B3	2 × 2	39.11 ± 0.12	5.33 ± 1.53
B4	2 × 2	39.63 ± 0.53	4.67 ± 0.58
C1	2 × 2	40.56 ± 0.63	127.33 ± 5.16
C2	2 × 2	40.67 ± 0.79	95.47 ± 24.61
C3	2 × 2	39.22 ± 0.18	72.60 ± 14.40
C4	2 × 2	39.56 ± 0.47	90.07 ± 28.60
D1	2 × 2	40.34 ± 0.42	123.87 ± 2.02
D2	2 × 2	39.31 ± 0.25	120.00 ± 4.58
D3	2 × 2	40.45 ± 0.59	62.40 ± 2.88
D4	2 × 2	39.50 ± 0.27	64.73 ± 3.31

## Data Availability

The original contributions presented in this study are included in the article. Further inquiries can be directed to the corresponding author.

## References

[1] Panda P.K., Sadeghi K., Seo J. (2022). Recent Advances in Poly (vinyl alcohol)/Natural Polymer-Based Films for Food Packaging Applications: A Review. Food Packag. Shelf Life.

[2] Branco A.C., Oliveira A.S., Monteiro I., Nolasco P., Silva D.C., Figueiredo-Pina C.G., Colaço R., Serro A.P. (2022). PVA-Based Hydrogels Loaded with Diclofenac for Cartilage Replacement. Gels.

[3] Rahmadiawan D., Abral H., Shi S.C., Huang T.T., Zainul R., Nurdin H. (2023). Tribological Properties of Polyvinyl Alcohol/Uncaria gambir Extract Composite as Potential Green Protective Film. Tribol. Ind..

[4] Shekaryar H., Norouzbahari S. (2024). A Review on Versatile Applications of Polyvinyl Alcohol Thin Films, Specifically as Sensor Devices. Polym. Eng. Sci..

[5] Cortes-Morales E.C., Rathee V.S., Ghobadi A., Whitmer J.K. (2021). A Molecular View of Plasticization of Polyvinyl Alcohol. J. Chem. Phys..

[6] Zhang N., Yang L., Pan H., Xing S., Li J., Song H., Zhou D., Ma C., Liu H. (2025). Hydroxyl-Crosslinked Polyvinyl Alcohol Films Reinforced with Nanocellulose and Tea Polyphenols for Antibacterial Preservation of Dried Tofu. Int. J. Biol. Macromol..

[7] Chen Y., Li J., Lu J., Ding M., Chen Y. (2022). Synthesis and Properties of Poly(vinyl alcohol) Hydrogels with High Strength and Toughness. Polym. Test..

[8] Tang Q., Chen C., Li M., Yue X., Liu X., Li H. (2025). High Barrier Composite Films Prepared from Polyvinyl Alcohol/Lignin/Montmorillonite Blends. Ind. Crops Prod..

[9] Lv C., Liu D., Tian H., Xiang A. (2020). Non-Isothermal Crystallization Kinetics of Polyvinyl Alcohol Plasticized with Glycerol and Pentaerythritol. J. Polym. Res..

[10] Fei W., Wu Z., Cheng H., Xiong Y., Chen W., Meng L. (2023). Molecular Mobility and Morphology Change of Poly(vinyl alcohol) (PVA) Film as Induced by Plasticizer Glycerol. J. Polym. Sci..

[11] Yazdi J.S., Salari M., Ehrampoush M.H., Bakouei M. (2024). Development of Active Chitosan Film Containing Bacterial Cellulose Nanofibers and Silver Nanoparticles for Bread Packaging. Food Sci. Nutr..

[12] Channa I.A., Ashfaq J., Siddiqui M.A., Chandio A.D., Shar M.A., Alhazaa A. (2022). Multi-Shaded Edible Films Based on Gelatin and Starch for Packaging Applications. Polymers.

[13] Praseptiangga D., Sesari A.R., Rochima E., Muhammad D.R.A., Widyaastuti D., Zaman M.Z., Syamani F.A., Nazir N., Joni I.M., Panatarani C. (2024). Development and Characterization of Semi-Refined Iota Carrageenan/Fish Gelatin-Based Biocomposite Film Incorporated with SiO_2_/ZnO Nanoparticles. Int. J. Biol. Macromol..

[14] Sangnim T., Sriamornsak P., Theerawatcharothai S., Fugnoot K., Wattanathammanon P., Vongsak B., Huanbutta K. (2022). Development of Fast-Dissolving Orodispersible Films Loaded with Cannabis Extract. Sci. Eng. Health Stud..

[15] Patomchaiviwat V., Sriamornsak P., Chansiri G., Limmatvapirat S., Supawattanakul A., Chonganon T., Keattiteerachai A., Piriyaprasarth S. (2022). Development of Edible Bubbles of Calcium Alginate for Encapsulating Energy Drinks. Sci. Eng. Health Stud..

[16] Bizymis A.P., Giannou V., Tzia C. (2023). Contribution of Hydroxypropyl Methylcellulose to Composite Edible Films and Coatings Properties. Food Bioprocess Technol..

[17] Kumar L., Ramakanth D., Akhila K., Gaikwad K.K. (2022). Edible Films and Coatings for Food Packaging Applications: A Review. Environ. Chem. Lett..

[18] Kumari N., Bangar S.P., Petrů M., Ilyas R.A., Singh A., Kumar P. (2021). Development and Characterization of Fenugreek Protein-Based Edible Film. Foods.

[19] Sriamornsak P., Kennedy R.A. (2006). A Novel Gel Formation Method, Microstructure and Mechanical Properties of Calcium Polysaccharide Gel Films. Int. J. Pharm..

[20] Piyawatakarn P., Limmatvapirat C., Sriamornsak P., Luangtana-Anan M., Nunthanid J., Limmatvapirat S. (2015). Effect of Glycerol on Properties of Tapioca Starch-Based Films. Adv. Mater. Res..

[21] Melikoğlu A.Y., Hayatioğlu N., Hendekçi M.C., Tekin İ., Ersus S. (2022). Development and Characterization of Edible Films Based on Carboxymethyl Cellulose Enriched with Pomegranate Seed Oil and Coating of Strawberries. J. Food Process. Preserv..

[22] Chen L.H., Doyle P.S. (2022). Thermogelling Hydroxypropyl Methylcellulose Nanoemulsions as Templates to Formulate Poorly Water-Soluble Drugs into Oral Thin Films Containing Drug Nanoparticles. Chem. Mater..

[23] Alipoori S., Aboutalebi S.H., Barsbay M. (2024). Enhancing the Performance of Solid-State Supercapacitors: Optimizing Molecular Interactions in Flexible Gel Polymer Electrolytes. J. Solid State Electrochem..

[24] Dzulhijjah W.A., Fitriani F., Aprilia S., Arahman N., Bilad M.R., Rahmah K., Akbar E.H., Raqib M. (2024). Formulation Optimization of Bionanocomposite Films Based on Polyvinyl Alcohol, Glycerol, and Cellulose Nanocrystals from Pineapple Crown Leaf Fibers Using Response Surface Methodology. IOP Conf. Ser. Earth Environ. Sci..

[25] Razmgar K., Nasiraee M. (2022). Polyvinyl Alcohol-Based Membranes for Filtration of Aqueous Solutions: A Comprehensive Review. Polym. Eng. Sci..

